# How Does Light Regulate Mood and Behavioral State?

**DOI:** 10.3390/clockssleep1030027

**Published:** 2019-07-12

**Authors:** Nina Milosavljevic

**Affiliations:** Faculty of Biology, Medicine and Health, University of Manchester, Manchester M13 9PL, UK; nina.milosavljevic@manchester.ac.uk

**Keywords:** light, mood, ipRGCs, non-visual responses, alertness, depression, behavioiral state

## Abstract

The idea that light affects mood and behavioral state is not new. However, not much is known about the particular mechanisms and circuits involved. To fully understand these, we need to know what properties of light are important for mediating changes in mood as well as what photoreceptors and pathways are responsible. Increasing evidence from both human and animal studies imply that a specialized class of retinal ganglion cells, intrinsically photosensitive retinal ganglion cells (ipRGCs), plays an important role in the light-regulated effects on mood and behavioral state, which is in line with their well-established roles in other non-visual responses (pupillary light reflex and circadian photoentrainment). This paper reviews our current understanding on the mechanisms and paths by which the light information modulates behavioral state and mood.

## 1. Introduction

Light is a powerful multifaceted stimulus; aside from supporting vision, light also affects a range of physiological and behavioral aspects. Well established among the so-called non-visual (NV) responses are circadian photoentrainment, pupillary light reflex and light regulation of sleep propensity. However, in the last decade, there has been growing evidence that light responsiveness extends to fundamental aspects of mood, behavior and performance. Functional imaging and psychometric assessments indicate that light can affect mood, alertness/attention and cognitive performance in **humans** [1]. These effects are typically described as being most effectively elicited by ‘brighter’ and ‘bluer’ lights and have thus been described first as primarily responses to changes in ambient illumination, and second, as likely to originate with a particular class of retinal neurons so-called intrinsically photosensitive retinal ganglion cells (ipRGCs). ipRGCs have the extraordinary capacity of responding to light directly thanks to their expression of the photopigment melanopsin (as well as performing the conventional ganglion cell function of acting as a conduit for signals originating in rods and cones). Recent studies in **mice** have started to elucidate the mechanisms and circuits for mediating these light effects driven by ipRGCs. 

Understanding how light affects mood has a direct practical implication on the health and well-being of people, especially now more than ever given that people spend most of their time indoors exposed to artificial lighting of various qualities that can be controlled. Moreover, useful new perspectives on the control of mood by understanding the mechanisms of light input may gain deeper insights into the general control of mood and reveal new therapeutic approaches for mood disorders.

## 2. Non-Conventional Retinal Ganglion Cells—ipRGCs

For decades, rods and cones were thought to be the only light detectors in the mammalian retina. The discovery of a third class of photoreceptors started when mice lacking conventional photoreceptors due to the naturally occurring rd/rd mutation were found to be still capable of circadian photoentrainment [2,3,4]. In addition, it was shown that rodless and coneless mice retained a pupillary light reflex [5] and regulation of pineal melatonin synthesis [6]. Finally, a small group of retinal ganglion cells (RGCs) that innervate the suprachiasmatic nucleus (SCN) was identified to be directly light responsive [7,8] thanks to the photopigment melanopsin [9,10]. Accordingly, triple knock out mice (mice lacking rods, cones, and melanopsin) were shown to be blind and incapable to photoentrain or constrict their pupils [11]. This new class of melanopsin-expressing RGCs was named as intrinsically photosensitive retinal ganglion cells (ipRGCs).

ipRGCs represent a small fraction of the total RGC population, comprising <5% of all RGCs. ipRGCs are not a uniform population of cells as first thought. Currently, there are six known subtypes of ipRGCs, M1–M6, primarily identified according to differences in their morphology and the brain regions they project to [12,13]. M1 was the first subtype to be characterized, with the highest level of melanopsin expression and therefore highest sensitivity to light. They are the smallest, and their dendrites stratify in the outer inner plexiform layer (IPL) (also known as the OFF sublamina of the IPL). M2, M4, and M5 cells are larger and stratify in the inner (ON) sublayer of the IPL. M3 cells are bistratified with dendrites in both the ON and OFF sublamina of the IPL [12]. The M1 subtype of ipRGC can be further divided based on the expression of the basic helix–loop–helix transcription factor (Brn3b). Brn3b, a POU domain transcription factor (also known as POU4F2), has important roles in RGC differentiation and axonal outgrowth as well as survival [14,15], and is used for cell type specification within the RGC population [16]. In contrast, M2–M5 cells all express Brn3b. Recently discovered M6 cells are small-field bistratified photosensitive Cdh3-GFP+ cells found in a mouse model where the expression of GFP (green fluorescent protein) is under the control of the promoter for cadherin-3 [13]. 

ipRGC subtypes also differ in the brain areas they project to, and therefore the functions they support. M1 cells project to the suprachiasmatic nucleus (SCN), the shell of the olivary pretectal nucleus (OPN), the intergeniculate leaflet (IGL), and the lateral geniculate nucleus (vLGN) as well as to limbic and other nonvisual thalamic nuclei such as the medial amygdala and peri-habenular region (pHb) [17]. Non-M1 ipRGCs project to the SCN, but also to the core of the OPN, to mood- and pain-modulating regions such as the periaqueductal gray (PAG) and amygdala, and to traditionally visual brain areas such as the dorsal LGN (dLGN) and the superior colliculus (SC) [12,18,19,20,21]. Brn3b-positive M1 cells project to the OPN and are essential for driving the pupillary light reflex [22]. Brn3b-negative M1 ipRGCs project to the SCN and are enough to drive circadian photoentrainment [22]. In addition, the ventrolateral preoptic nucleus (VLPO) is a hypothalamic area implicated in sleep induction, which receives input only from Brn3b-positive ipRGCs [23]. M6 projections are found in dLGN, while their projections in OPN, IGL, and vLGN are shared with the M5 subtype [13].

## 3. Circadian Rhythm and Mood 

The most potent impact of light on physiology and behavioral state comes from its regulation of circadian rhythms. Mammalian physiology and behavior are coordinated by circadian clocks into rhythms that are synchronized with the light–dark cycles of a 24-hour solar day. These molecular clocks, based on interlocked transcription/translation feedback loops, are present in most cells throughout the body and are synchronized by a ‘master’ clock located in the suprachiasmatic nucleus (SCN) of the hypothalamus [24]. This endogenous clock is self-sustained even in the absence of external stimuli, but can be entrained to light–dark cycles in a process called photoentrainment.

A change in the timing of the light–dark cycle (e.g., light exposure at night time) will result in a phase shift of circadian rhythms. Many behavioral and physiological functions such as sleep–wake regulation, hormone secretion, and thermoregulation vary across the light–dark cycle coordinated with the circadian rhythm, therefore, it is not surprising that a disruption of circadian rhythm can impact gross physiology and behavior. 

For years, there has been a clear connection between circadian rhythms and mood. In fact, most people who suffer from mood disorders are shown to have significant disruptions in their circadian rhythms and the sleep/wake cycle [25,26,27]. Environmental disruptions to circadian rhythms such as shift work, travel across time zones, and altered sleep–wake cycles tend to have strong effects on mood and mental illness [28,29,30]. There could be more than one way in which circadian disruption affects mood. SCN and the circadian genes are involved in the regulation of immune function, monoamine transmission, neurogenesis, and metabolism [31]. It is also possible that circadian rhythms can affect mood and the behavioral state via SCN connections to the mood regulatory centers. The SCN receives direct input from the retina and then sends signals via direct and indirect projections throughout the brain to various regions involved in the regulation of mood and behavioral state including the paraventricular nucleus of the hypothalamus (PVN) [32], dorsomedial hypothalamus [33], paraventricular nucleus of the thalamus [34,35], arcuate nucleus [36], bed nucleus of the stria terminalis [37], lateral habenula [38], locus coeruleus [39,40], and the dorsal raphe [27,41]. The SCN also regulates the hypothalamo-pituitaryadrenal (HPA) axis via PVN [42]. Circadian disruptions are often accompanied by HPA axis dysregulation, and glucocorticoid hormones have complex effects on many systems including the brain and the immune system [43,44]. 

## 4. Direct Effects of Light on Mood and Behavioral State

Although circadian clocks are self-sustained, a change in the timing of the light exposure will result in a phase-shift of circadian rhythms. Therefore light can indirectly affect overall physiology and behavioral state. However, light can also have more direct effects on physiology and behavior. Recent studies in **humans** showed that light could trigger physiological changes (the heart, and thermoregulation) within a minute of exposure [45], while functional imaging revealed the effects of light on the limbic structures (amygdala and hippocampus) within seconds of exposure [1,46]. Moreover, growing evidence suggests that artificial light exposure at night time and/or particular spectral compositions of the light have direct effects on mood and behavioral state. Functional imaging and psychometric assessments in both the laboratory and field indicate that light can affect mood, alertness/attention, waking EEG, and cognitive performance in people [1,46,47,48,49,50,51,52,53,54,55,56,57,58,59]. 

### 4.1. Melanopsin and ‘Blue’ Light 

**Figure 1:**
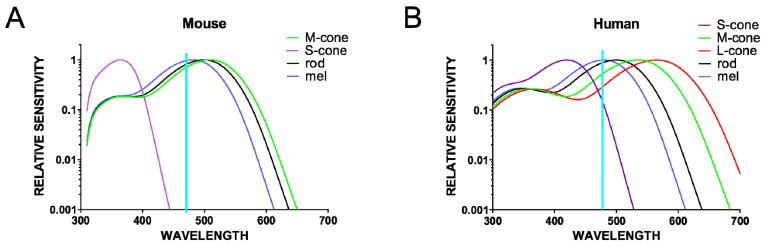
Relative sensitivity curves for all of the opsins present in the mouse (**A**) and human (**B**) retina. Note the limitation of using blue light (~470 nm) to excite melanopsin only (λ_max_~480 nm); although there is significant overlap with the melanopsin peak sensitivity, this light also excites other photoreceptors to different extents.

With the discovery of ipRGCs and their function in non-visual (NV) responses (pupillary light reflex, circadian photoentrainment, and sleep propensity), the idea that these photoreceptors could drive other NV responses arose. To test this hypothesis, the effects of monochromatic lights were examined, particularly that of the ‘blue’ light. The rationale behind using the blue monochromatic light was that melanopsin, a photopigment of ipRGCs, has a peak sensitivity in the blue spectrum of the visible light (479 nm for human and 484 nm for mouse melanopsin) [60]. In addition, a monochromatic light in the green spectrum is often used as the excitatory for conventional photoreceptors (medium wavelength cones, M-cones have a peak sensitivity at 508 nm in mice and 535 nm in humans; and long wavelength cones; L-cones have a peak sensitivity at 565 nm in humans [61], Figure 1). Studies in **humans** when comparing the effects of blue (460 nm) and green (550/555 nm) light exposures have revealed greater responses to ‘blue’ light for acute suppression of melatonin secretion, increase of body temperature and heart rate, and the reduction of subjective sleepiness with an increase in alertness [55,57]. These studies thus suggest that blue light (460 nm), which better activates ipRGCs, has more alerting effects than green light (550/555 nm) [62].

**Figure 2:**
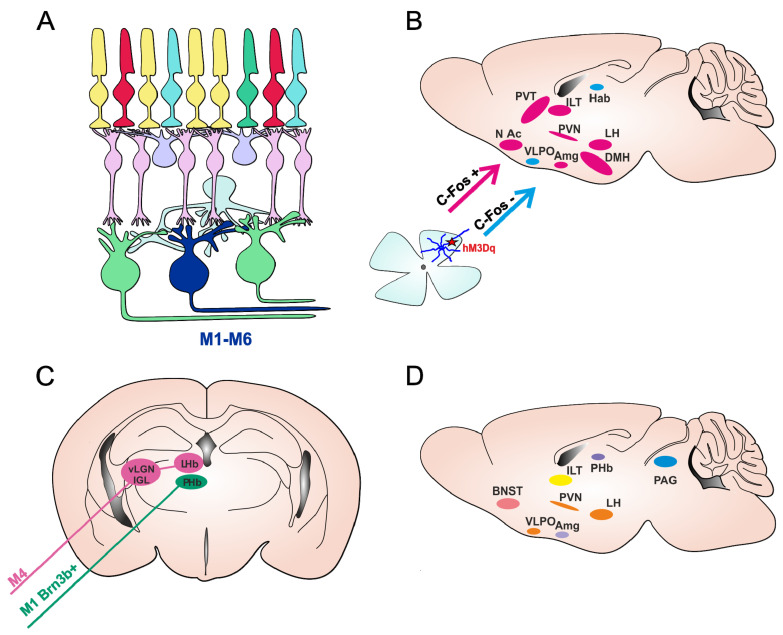
ipRGC pathways for mood regulation in mice. (**A**) Schematic presentation of mammalian retina, with six subtypes of ipRGCs (M1–M6). (**B**) Brain areas involved in the regulation of mood and behavioral state were shown to have increased activity (measured upregulation of c-Fos, an early marker of neural activation) following the acute activation of ipRGCs using chemogenetics (ipRGCs expressing Gq-coupled chemogenetic hM3Dq receptor, depicted by a red star), in pink. Nuclei labelled in blue showed no c-Fos increase. Figure adapted from [63]. (**C**) Two identified pathways by which ipRGCs are involved in light-induced regulation of depressive-like behavior. The M1 Brn3b+ ipRGCs project directly to PHb (perihabenular nucleus) and are involved in depressive-like behaviors in mice triggered by chronic aberrant light exposure (T7 cycle). M4 ipRGCs innervate the vLGN/IGL-LHb (ventral lateral geniculate nucleus/intergeniculate leaflet-lateral habenula) pathway and are responsible for short-term light effects in decreasing depressive-like behavior. (**D**) Nuclei involved in the regulation of mood and behavioral state that receive direct projections from ipRGCs. *Paraventrical hypothalamic nucleus (PVN), the dorsomedial hypothalamus (DMH), lateral hypothalamus (LH), ventrolateral preoptic nucleus (VLPO); amygdala (Amg), intralaminar thalamic nuclei (ITL), paraventricular thalamus (PVT), lateral habenula (LHb), nucleus accumbens (NAc), Bed nucleus of the stria terminalis (BNST) and periaqueductal gray (PAG)*.

Although **mice** are nocturnal animals, unlike humans who are diurnal, multiple lines of evidence suggest that light can also have alerting effects on mice [63,64]. Mouse models are invaluable assets for studying light-mediated effects on behavioral state to allow the assessing of particular circuits and identifying mechanisms of action with the development of new tools and technology for manipulating distinct cell types. Using light to selectively activate a particular photoreceptor class has intrinsic limitations as light will inevitably also activate other photoreceptor classes (Figure 1 and Figure 2A). One way to exclusively activate ipRGCs is with chemogenetics. Chemogenetics have been successfully used to address specific questions about the roles of ipRGCs in visual and NV responses [63,65,66,67]. In a light-independent study, the Gq-coupled chemogenetic tool hM3Dq, which reliably induces the depolarization of neurons upon the administration of clozapine N-oxide [CNO] [68], was expressed only in ipRGCs. This allowed for the mimicking of the excitatory effect of light for ipRGCs without producing any other visual experience and thus stimulating any other photoreceptors in the retina. This selective activation of ipRGCs induced alerting and anxiety-like behavior in mice, similar to the effects of bright light on wild type mice [63]. Further demonstration that this response is retained in rodless and coneless mice and is lost in melanopsin knockouts confirmed that an increase in alertness is a natural light response for which ipRGCs are both necessary and sufficient [63]. Behavioral results are in agreement with c-Fos mapping performed after the chemogenetic activation of ipRGCs, which revealed the activation of brain regions involved in the regulation of mood and behavioral state, typically active during wakefulness and arousal (Figure 2B) [63].

Therefore, the simplest current model would be that changes in behavioral state are elicited by increases in ambient illumination encoded by ipRGCs. However, there is also evidence that the situation is more complex. Pilorz and colleagues reported that while blue light (~470 nm) increased arousal, green light (530 nm) induced sleep in **mice** [64]. This remarkable wavelength dependence was interpreted in terms of the differences in the efficiency with which the various mouse photoreceptors responded to light at these two wavelengths (Figure 1). Thus, as cones are more sensitive than melanopsin to 530 nm, one interpretation of these data is that cones drive sleep and melanopsin drives arousal. However, alternative possibilities are that responses to both stimuli originate with melanopsin, with ‘dimmer’ light (in this case represented by the less favored wavelength) producing sleep and brighter light, arousal; or with cone-based assessments of stimulus color [69]. In any event, the observation that visual stimuli can have quite opposite behavioral effects matches previous evidence that light can enhance sleep in mice [70].

### 4.2. Divergent Neural Pathways Link ipRGCs to Mood 

Our current knowledge implies that light can drive different influences on mood and behavioral state dependent on the qualities of light, but also on the context of immediate or long-term effects. A range of studies in **mice** have used various approaches from different light stimulation (wavelength, irradiance) to chemogenetics, exploring the short or long-term effects. These different types of paradigm have resulted in different outcomes, suggesting that light could actually have multiple routes to mood regulation. 

Studies focusing on the long-term effects of light on mood have used the interrupted light–dark cycle, T7 cycle (3.5 h light and 3.5 h dark phase), which mimics aberrant light exposure as a result of shift-work or transmeridian travel. Long-term exposure to the T7 cycle has revealed a depressive state in **mice** associated with the activation of the lateral habenula (LHb) driven by ipRGCs [71]. The authors continued to further investigate these effects and recently identified a new pathway that drove the light-mediated depressive like behaviors from ipRGC directly to the perihabenular nucleus (ipRGCs-PHb) (Figure 2C) [72].

Interestingly, these depressive-like behaviors were not detected in studies investigating the short-term effects of ipRGCs through using chemogenetics [63] or light [73]. In fact, the short-term activation of ipRGCs tended to decrease depressive-like behavior, but increased arousal in **mice** [63]. Moreover, a recent study identified another pathway in which the M4 subtype of ipRGCs innervated neurons in the thalamic ventral lateral geniculate nucleus and intergeniculate leaflet (vLGN/IGL), which in turn inhibit neurons in the LHb (Figure 2C). The authors further showed that the activation of this retina-vLGN/IGL-LHb pathway was responsible for decreasing depressive-like behavior and for the light effects of light therapy on depressive symptoms [73]. 

Studies in mice on the long-term effects of light involving the interrupted LD cycle (T7) could have a more pronounce effect on overall physiology and sleep, and sleep disturbance can directly have effects on mood. Therefore, it may not be surprising that the outcome of studies with a T7 cycle are different from studies investigating the acute effects of light in animals exposed to a normal LD cycle. Regarding the mechanisms involved, it is possible that during the T7 cycle, both subpopulations of ipRGCs (the pHb-projecting ipRGCs that are mostly M1-type and M4-type ipRGCs) were activated, but that the chronic activation of the retina-pHb was a dominant pathway causing depressive effects. Moreover, these pathways may not be exclusive for mediating light-induced influences on mood. ipRGCs also directly project to other areas involved in mood and behavioral state regulation such as the amygdala, bed nucleus of the stria terminalis, PVN, and periaqueductal gray [18,19] (Figure 2D).

### 4.3. Beyond ‘Blue’ Light

Most studies in **humans** have used monochromatic blue light with the idea to reveal melanopsin and thus ipRGC involvement in various NV responses. Although, it is true that the ‘blue’ light used is close to the peak sensitivity of melanopsin, it will still activate other photoreceptors in a mammalian retina to various extents including M-cones, L-cones, and rods (Figure 1). Therefore, outcomes from studies with ‘blue’ light do not exclude the involvement of other photoreceptors in mood regulation. In fact, rods and cones have already been shown to be involved in some NV responses including the pupillary light reflex and circadian photoentrainment. From studies in **mice**, we learnt that while these NV responses persist in the absence of rods and cones [3,4,5,6], thus demonstrating a critical role of melanopsin, the loss of melanopsin does not abolish these responses [74,75]. In contrast, the loss of ipRGCs produces a dramatic loss of these NV responses, [70,76] emphasizing that ipRGCs are not solely photoreceptors, but as ganglion cells, also provide an important conduit for signals the from rods and cones. 

Increasing evidence suggests that information from conventional photoreceptors is also important in the regulation of NV responses such as circadian entrainment and pupillary light reflex [69,77,78]. The color of light is also a signal for photoentrainment of the circadian clock in **mice** as well as for pupillary light reflex, directly implying the involvement of cones in these NV responses [69,78]. It is most likely that ipRGCs provide this color information as they are the major retinal input to the suprachiasmatic nucleus (SCN) and the olivary pretectal nucleus (OPN). Accordingly, it has been shown that these ganglion cells do receive input from the rods and cones in **mice** [79]. Moreover, a morphologically and functionally distinct ipRGC subtype (M5 cells) was shown to respond to both melanopsin and chromatically opponent cone-based signals (UV-ON, green-OFF) [80]. Although it seems M5 cells project to the visual thalamus (dLGN) and are therefore involved in visual responses, the possibility that they are involved in some NV responses is yet to be excluded. These recent studies in **mice** therefore imply that ipRGC involvement in the light effects on mood and behavioral state could, in addition to melanopsin, also be driven by rods and cones.

## 5. Conclusions

The effects of light on mood and behavioral state are not only mediated by melanopsin as first thought. Based on our recent knowledge from **mouse** models on NV responses including the pupillary light reflex and circadian photoentrainment, it is possible that inputs from the rods and cones play roles in driving the light effect on mood and behavioral state. The spectral composition of light as well as the duration of exposure could have various and even opposing effects on one’s mood and behavioral state. While there is growing evidence from both **mice** and **human** studies that light stimuli can influence mood and behavioral state, we still do not know what light features are salient for these systems. Is this only a change in ambient light (irradiance), or does it also involve changes in color? Additionally, what about the time of day when light stimulation is presented as well as its duration? Finally, whether and how these parameters differ for **mice** and **humans**? More systematic studies are needed to further deepen our knowledge in which light features regulate mood and behavioral state. 

Studies in **humans**, although crucial for studying mood, have intrinsic limitations in the experimental design for the selective activation of a particular photoreceptor type, they rely on a careful design of light conditions. Monochromatic light, even with a wavelength matched to the peak spectral sensitivity of a given photoreceptor, will stimulate all photoreceptor classes, making it difficult to dissect the pathways of mechanism driven by a particular photoreceptor type involved. However, there is a way to selectively stimulate a particular class of photoreceptors while keeping the activation of all others consistent, the so-called method of silent substitution. This approach uses pairs of light stimuli (metamers) that have the same color and luminance, but different spectral power distribution. Metamers are designed to have balanced changes in the intensity of various wavelengths of light in order to stimulate a particular photoreceptor, but without altering the effective photon flux for other classes of photoreceptors. This method has been used successfully to investigate melanopsin involvement in visual processing in **mice** [81,82,83,84,85] and the photoreceptor contributions to the pupillary light reflex in **humans** [86,87,88,89]. Allen and colleagues built a visual display capable of presenting metameric images differing in melanopsin effective irradiance to **humans**. This bespoke visual device allowed them to test the ability of melanopsin to control NV responses such as melatonin onset and self-reported alertness in the context of a typical screen use [90]. 

While studies in **humans** have obvious benefits in studying aspects of mood, studies in **animal** models are equally important as they allow for the determination of the particular circuits and mechanisms involved. Knowledge acquired from **animal models** on mood and behavioral state, however, needs to be carefully interpreted and not automatically extrapolated to humans because: (i) studies in rodents are done in nocturnal species that are photophobic and therefore light as a stimulus may not always have the same effect as in humans who are diurnal; and (ii) an obvious limitation when studying mood in animals is how to mimic the mood and mood disorders humans experience in rodent models. The former could perhaps be addressed by using diurnal rodents such as the Nile grass rat (*Arvicanthis niloticus*) [91], while the latter is a challenge that all mood-related studies face and thus great efforts are being invested in developing better animal models and behavioral assessments. Nevertheless, recent studies in **mice** have shed light on some of the circuits involved in the light-driven regulation of mood, but have also indicated their complexity, as the same photoreceptor class (ipRGCs) is suggested to drive even opposing effects on mood depending on the context. Up to date literature implies the importance of ipRGCs in driving NV responses (from the pupillary light reflex, circadian photoentrainment to mood and behavioral state), but exciting new evidence suggests that ipRGCs not only mediate signals from melanopsin as a response to ‘blue’ light, but also carry signals from other photoreceptors. Future studies will aim to provide further evidence for which photoreceptor classes and light parameters (irradiance, color, duration of exposure, and time of the day) play roles in mood and behavioral state. This information will be of immense value for artificial lighting design to maximize people’s health and wellbeing in indoor spaces as well as for light therapy for patients in need. Seasonal affective disorder (SAD) is a type of depression that occurs during the same season every year, usually in late autumn or winter when the daylength becomes shorter. The spectral composition of natural light varies with the seasons [92], and, together with the total light irradiance, is thought to contribute to SAD. Light therapy is the treatment of choice for SAD, but it is also emerging as a promising treatment for many other psychiatric disorders including depressive discords and bipolar depression [93]. Cognitive improvement under light therapy has been noted in adult attention deficit hyperactivity disorder [94]. In order to exploit the full potential of light therapies, we need to have a better understanding on how they work and how they affect our brain and behavioral state.
